# Whole-Body Radioiodine Effective Half-Life in Patients with Differentiated Thyroid Cancer

**DOI:** 10.3390/diagnostics11101740

**Published:** 2021-09-22

**Authors:** Michele Klain, Carmela Nappi, Marina De Risi, Leandra Piscopo, Fabio Volpe, Mariarosaria Manganelli, Elisa Caiazzo, Davide Bianco, Martin Schlumberger, Alberto Cuocolo

**Affiliations:** 1 Department of Advanced Biomedical Sciences, University Federico II, 80131 Naples, Italy; micheleklain@libero.it (M.K.); c.nappi@unina.it (C.N.); eagletea16@gmail.com (M.D.R.); lea-17-08@hotmail.it (L.P.); fabiovolpe84@outlook.it (F.V.); mariarosaria.manganelli.87@gmail.com (M.M.); elisa_caiazzo@hotmail.it (E.C.); Martin.SCHLUMBERGER@gustaveroussy.fr (M.S.); 2 Italian Aerospace Research Centre (CIRA), 81043 Capua, Italy; davidebianco1@gmail.com

**Keywords:** iodine, radionuclide therapy, thyroid cancer

## Abstract

**Background**: Radioactive ^131^I (RAI) therapy is used in patients with differentiated thyroid cancer (DTC) after total thyroidectomy for remnant ablation, adjuvant treatment or treatment of persistent disease. ^131^I retention data, which are used to indicate the time at which a ^131^I treated DTC patient can be released from the hospital, may bring some insights regarding clinical factors that prolong the length of hospitalization. The aim of this study was to investigate the ^131^I whole-body retention in DTC patients during ^131^I therapy. **Methods**: We monitored 166 DTC patients to follow the ^131^I whole-body retention during ^131^I therapy with a radioactivity detector fixed on the ceiling of each protected room. A linear regression fit permitted us to estimate the whole-body ^131^I effective half-life in each patient, and a relationship was sought between patients’ clinical characteristics and whole-body effective ^131^I half-life. **Results:** The effective ^131^I half-life ranged from 4.08 to 56.4 h. At multivariable analysis, longer effective ^131^I half-life was related to older age and extensive extra-thyroid disease. **Conclusions:** ^131^I effective half-life during ^131^I treatment in DTC patients is highly variable among patients and is significantly longer in older and in patients with RAI uptake in large thyroid remnants or in extrathyroidal disease that significantly prolongs the whole-body retention of ^131^I.

## 1. Introduction

Radioactive ^131^I (RAI) therapy is used in patients with differentiated thyroid cancer (DTC) after total thyroidectomy for remnant ablation, adjuvant treatment or treatment of known persistent disease. The efficacy of RAI therapy has been related to the radiation dose delivered to normal and neoplastic thyroid tissues [1,2,3]. Yet, the delivery of significant radiation doses to extrathyroidal tissues may induce side effects [4,5,6,7]. Hence, treatment conditions should optimize the radiation dose delivered to thyroid tissues and minimize the radiation dose delivered to extrathyroidal tissues. ^131^I uptake in thyroid cells is related to cell differentiation and to the intensity of stimulation with thyroid-stimulating hormone (TSH) [8] in the absence of iodine contamination. The radiation doses delivered to normal and neoplastic thyroid tissue can be estimated by lesion dosimetry from the initial uptake and effective ^131^I half-life in the thyroid tissue [9,10,11,12]. To estimate doses absorbed by extrathyroidal tissues, the Medical Internal Radiation Dose formalism is generally adopted [13]. Data published in report 53 of the International Commission on Radiological Protection can be used for dose estimates in healthy subjects [14]. However, these data may be irrelevant in patients with DTC, for the following reasons: during hypothyroidism after prolonged thyroid hormone withdrawal, radioiodine renal clearance is decreased, prolonging its body retention. In fact, in euthyroid thyroid cancer patients treated with recombinant human TSH (rhTSH), mean ^131^I effective half-life is shorter by 31% than in hypothyroid patients undergoing thyroid hormone withdrawal [15,16]; the activity of the high amounts of radioiodine used for therapy may have a biodistribution different from that observed with lower activities used for diagnosis, the morphology may vary among patients, and uptake may be present in metastases. ^131^I retention studies might bring some insights into patients’ radionuclide kinetics and might indicate which patients might receive higher body radiation doses and for whom some caution is needed in determining the maximal activity that can be safely administered. In clinical practice, retention data were used to indicate the time at which a thyroid cancer patient treated with ^131^I could be released from the hospital. Under Italian law, a dose rate <30 μSv/h at 1 m, approximately corresponding to a whole-body retention of <600 MBq, permits patients to be discharged from the hospital. In this study, we analyzed the whole-body effective ^131^I half-life in DTC patients undergoing RAI treatment and sought for factors that might prolong it.

## 2. Materials and Methods

### 2.1. Patients

A total of 166 DTC patients referred to our department for RAI therapy were enrolled. Patients were categorized according to the age cut-off of 55 years in agreement with 8th edition of the American Joint Committee on Cancer staging system for papillary thyroid cancer [17]. Low-iodine diet was recommended for 7–14 days before RAI administration: patients were asked to discontinue the use of iodine-containing preparations and medications for 3 weeks, and no water-soluble iodinated contrast medium was administered at least for 6 weeks before RAI treatment. Thyroid hormone (L-thyroxine) treatment was withdrawn for 3 weeks before RAI administration and until serum TSH reached an arbitrary level of 30 mIU/L; in patients with a TSH below this level, withdrawal was prolonged for 7 days and TSH level was measured again before RAI administration. Patients received oral and written information concerning ^131^I treatment and radioprotection before oral administration of ^131^I as capsules. According to the American Thyroid Association risk stratification categories of recurrence [18], the activity administered in 36 low-risk patients ranged from 1110 to 2220 MBq, in 119 intermediate risk patients from 2479 to 3700 MBq and in 11 high risk patients from 4440 to 7400 MBq. Patients were hospitalized for 2.5–3 days in protected rooms. Abundant hydration and laxative treatment were given during the hospitalization. Serum thyroglobulin (Tg), Tg-antibody (Tg-Ab), and TSH levels were obtained after withdrawal of L-thyroxine treatment one day before RAI administration. Neck ultrasound was performed before RAI treatment and a whole-body scan (WBS) was performed 7 days after ^131^I administration using a dual-head gamma camera equipped with thick crystals and high-energy collimators (Skylight, Philips Healthcare, Best, The Netherlands).

### 2.2. Monitoring System

**Figure 1:**
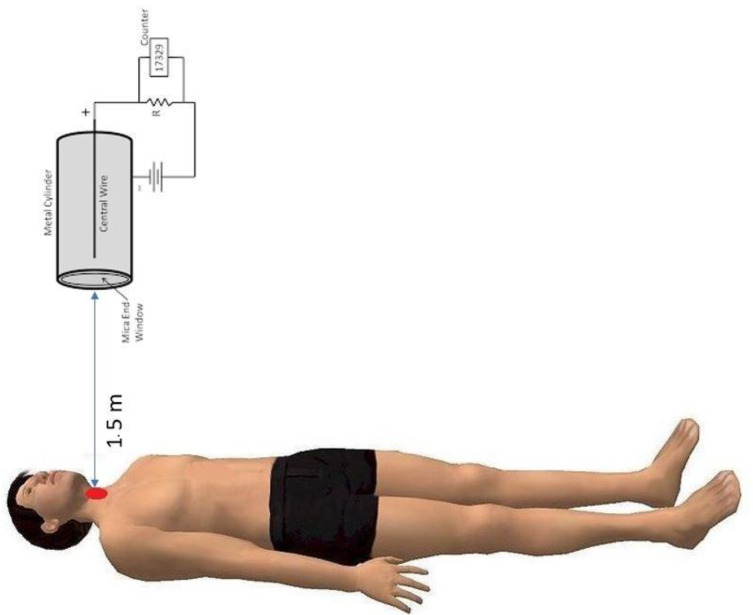
An illustration of the monitoring system used for this study. Data have been measured with a Geiger–Muller counter fixed on the ceiling of each protected room, at approximately 1.5 m above the patient’s head.

The whole-body ^131^I effective half-life was estimated using a Geiger–Muller counter fixed on the ceiling of each protected room at the head of each patient bed and connected to a measurement electronics controlled by a PC, allowing the acquisition of data on patient radioactive levels until discharge. The radiation-detector survey meter counter was calibrated for measuring the rate of H*(10) (μSv/h) (Figure 1). For each patient, all measurements were performed by the same probe and with the same distance (150 cm) between the ceiling and the bed. An alarm advised the patient when the measurement was going to start and stop. Measurements were performed at 2 h after ^131^I administration before any urinary elimination (a data point corresponding to 100% of the administered activity) and then every 2 h until 48 h after patients had emptied all urine from their bladder. The whole-body ^131^I effective half-life was defined as the time required for the amount of ^131^I estimated at 2 h following administration be divided by a factor of 2, we obtained a linear regression fit from the combined action of physical decay and biological disappearance [14,16] to estimate the whole-body ^131^I effective half-life in each patient.

### 2.3. Statistical Analysis

Continuous data are expressed as mean ± standard deviation and categorical data as percentage. Differences between groups were analyzed by unpaired t test or chi square test, as appropriate. Correlation analysis with Bravais–Pearson correlation coefficients analysis was used to assess the relationship between variables. Multivariable linear regression analysis was performed to evaluate the relationship between patients’ clinical characteristics and whole-body effective ^131^I half-life by using the backward elimination method. All clinical variables were put into the multivariable model, with a significance level of 0.1 for variables to stay in the model. The multivariable analysis has been then performed iteratively until all variables in the model showed p-values below the chosen level of 0.1. The percentage of explained variance (R2) was calculated to give an indication of the predictive power of the final multiple regression model. Only variables showing a *p* value < 0.05 were considered statistically independent. Statistical analysis was performed with STATA version 15.1 for Windows (StataCorp LP, College Station, TX, USA).

## 3. Results

Baseline characteristics of the 166 patients are reported in Table 1.

Among 166 patients, 89 (54%) were classified as T1, 56 (34%) were considered T2, 12 (7%) were considered as T3 and 9 (5%) were classified as T4 at histopathological examination. As described in Table 1, there were 33 (20%) patients showing metastases, 7 (4 %) patients had only lymph node involvement, 13 (8%) patients had distant metastases and 13 (8%) had both lymph node and distant metastasis. Median whole-body effective ^131^I half-life in the overall population was 13.7 h (range 4.1–56.4). Mean whole-body effective ^131^I half-life was significantly (*p* < 0.01) longer in patients with extra-thyroidal uptake at WBS (19.9 ± 12.2 h) compared to those without (15 ± 7.9 h). Of note, there were 9 patients without thyroid remnant uptake. The mean whole-body effective ^131^I half-life did not differ between patients with thyroid remnant uptake and those without (15.6 ± 9 h vs. 16.6 ± 10.9 h, *p* = 0.845) while the mean whole-body effective ^131^I half-life in patients showing both bone and lung metastases was 24.29 ± 12.8 h. In the overall population, the mean ^131^I whole-body retention at 48 h after RAI was 13.5 ± 11.9%.

**Figure 2:**
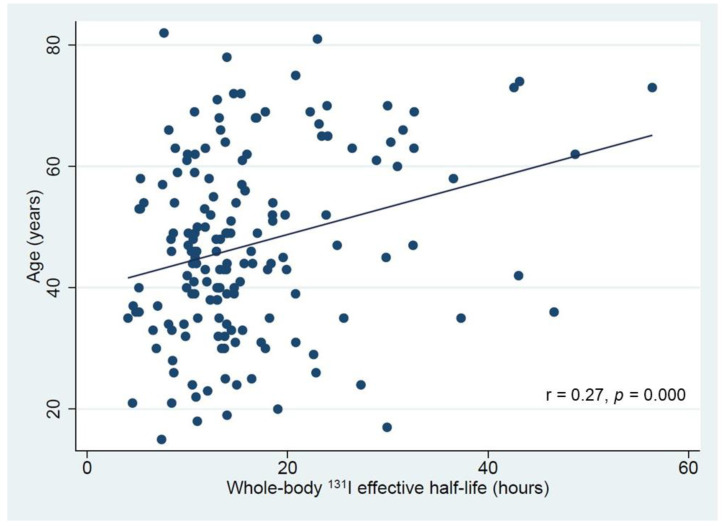
Relation between whole-body effective ^131^I half-life and age in patients with differentiated thyroid cancer.

As illustrated in Table 2, at multivariable linear regression analysis after backward variable selection was used to predict whole-body effective ^131^I half-life. While age, sex, extra-thyroidal disease and TSH off LT4 were included in the reduced model with a significance p value level <0.1, age and extra-thyroidal disease were independently associated with a longer effective ^131^I half-life *p* value 0.004 and 0.007, respectively. A weak albeit significant (r = 0.27, *p* = 0.0001) relationship between whole-body effective ^131^I half-life and age was also found (Figure 2).

**Figure 3:**
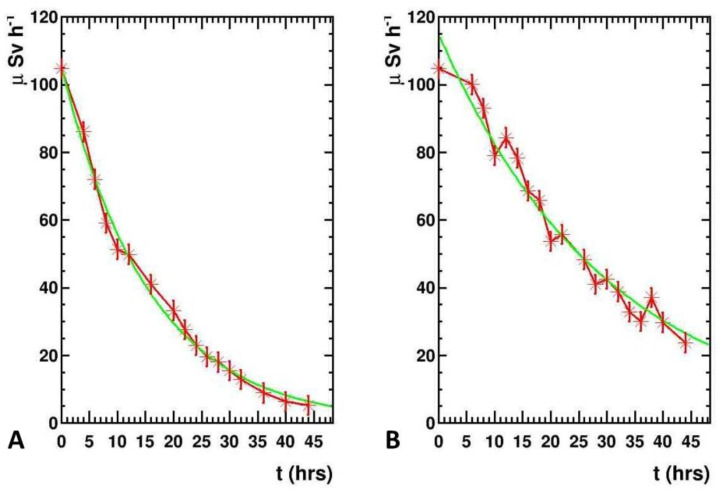
Effective ^131^I half-life regression curves for two patients, to whom the same initial activity (3700 MBq) was administered. (**A**) refers to a patient without ^131^I extra-thyroidal uptake at WBS and (**B**) refers to a patient with ^131^I extra-thyroidal uptake at WBS. The best fits to the experimental data were obtained using a mono-exponential (**A**) and a bi-exponential curve (**B**).

Whole-body effective ^131^I half-life has been obtained with a mono-exponential fit for patients without extra-thyroidal uptake at WBS, whereas a bi-exponential curve has been used for the others. In Figure 3, examples of effective ^131^I half-life regression curves for two patients, to whom the same initial activity (3700 MBq) has been administered, have been illustrated. 

Only a few datapoints were available at follow up for 133 out of the 166 patients (80%) with a mean follow up of 483 ± 60 months (range 2–232 months). Of these, 22 patients underwent a subsequent RAI therapy for persistent/recurrent disease. In these patients Tg levels on TSH stimulation at the time of the subsequent RAI therapy ranged from 4 to 4321 ng/mL. Among these, 3 patients also underwent neck lymph-node surgical resection, 3 patients showed lung metastases and one patient showed bone involvement at post-therapeutic WBS after the subsequent therapy. 

**Figure 4:**
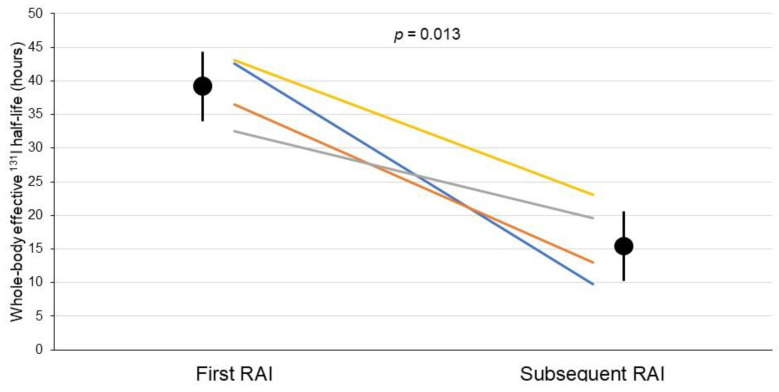
Effective ^131^I half-life levels measured at the time of first RAI and during the subsequent RAI treatment in the 4 patients with low serum TSH levels at the first treatment course. A significant difference was observed between effective ^131^I half-lives estimated at first and subsequent RAI treatment. Circles indicates effective ^131^I half-life mean values, lines describe effective ^131^I half-life range.

In 11 out of these 22 patients, the whole-body effective ^131^I half-life data were available for two consecutive courses of RAI therapy (Table 3). Four patients, after 3 weeks of LT4 withdrawal, had a low TSH level (≤7 mIU/L) at the time of the first RAI therapy and had significantly higher TSH levels (>30 mIU/L) at the time of the subsequent RAI therapy. The other 7 patients had TSH levels >30 mIU/L at the time of both RAI therapies after prolonged LT4 withdrawal for 3 weeks. Whole-body effective ^131^I half-life significantly decreased from the first to the second RAI course in the 4 patients with low serum TSH levels at the first treatment course (from 38.7 ± 5.1 to 16.4 ± 6.1 h, p_= 0.013, Figure 4) and also in 2 of the 7 patients with elevated serum TSH at both treatment courses. In these 6 patients, the uptake in the central neck was high at the first treatment course, indicating the presence of large thyroid remnants that were responsible for the relatively low serum TSH levels in 4 patients and were almost not visible at the second RAI treatment course. The effective ^131^I half-life was similar in the other 5 patients with elevated serum TSH n both treatment courses and who had visually less intense uptake in thyroid foci at the first treatment course.

## 4. Discussion

The results of the present study show that median whole-body effective ^131^I half-life in the overall population was 13.7 h (range 4.08–56.4), consistent with previous data, and was extremely variable among patients [15,19,20,21]. A longer whole-body effective ^131^I half-life was independently associated with older age and with the presence of extrathyroidal disease uptake. Kumar and coworkers [22] also investigated the ^131^I effective half-life in young DTC patients and from mono-exponentially curves obtained an overall median half-life of 36.6–90.27 h. However, they used a lesion-based approach, drawing regions of interest of each lesion on serial WBS at different time points under a single head gamma camera. This different method may explain discrepancies with our data [22]. Multiexponential regressions may give a reasonable physical interpretation because, under certain conditions, they represent multicompartmental models [23]. In other words, the number of different time constants to be employed for fitting experimental data is linked to the number of compartments in which the system can be divided. A biexponential fit is then more suitable for describing a patient with both thyroid remnant and metastases radioiodine uptake.

The relationship between ^131^I half-life and age might be related to a decrease in kidney function with ageing that might be even more relevant in hypothyroid conditions. Previous dosimetry studies have shown that ^131^I high activities might induce hematological toxicities in elderly patients due to prolonged ^131^I body retention and caution is recommended in these elderly patients [11]. In 11 patients, ^131^I body retention was further studied during another treatment course. In five of the seven patients undergoing both RAI treatments with elevated TSH levels, ^131^I whole-body effective half-life was similar. In the other two patients and in the four patients with low TSH levels at the first treatment course despite prolonged thyroid hormone withdrawal, the ^131^I effective half-life values at the first treatment course were significantly longer than during the subsequent RAI treatment course. This longer effective ^131^I half-life at the first treatment course might be related to the high uptake and prolonged RAI retention in normal and neoplastic thyroid foci, and this uptake was much lower at the second treatment course. Our findings are also consistent with findings in the literature findings [21,22] reporting that lesion ^131^I half-life was longer at first RAI therapy and decreased at subsequent courses of RAI therapy. This may explain in part the variable effective half-life duration observed among patients, a variability that was not reported in a previous study [15] in which thyroid remnants were either minimal or absent. Although our data regarding two therapy courses refer to a small number of patients, the results of the current investigation support the hypothesis that the presence of extra-thyroid uptake foci of RAI is associated with longer whole-body effective half-life. Analysis of whole-body effective ^131^I half-life differences between treatments in larger cohorts should be encouraged for a deeper understanding of ^131^I retention in patients undergoing more than one treatment course. Hence, a multicenter investigation may strengthen the value of the present findings. 

## 5. Conclusions

^131^I effective half-life during ^131^I treatment in patients with DTC is highly variable among patients and is significantly longer in older patients and in those with RAI uptake in large thyroid remnants or in extra-thyroidal disease.

## Figures and Tables

**Table 1:** Baseline characteristics in the overall population according to age.

	All Patients (*n* = 166)	<55 years (*n* = 117)	≥55 years (*n* = 49)	*p*
Age (years)	46.9 ± 14.9	39.2 ± 9.7	65.5 ± 6.5	0.0001
Male gender, *n* (%)	40 (24)	26 (22)	14 (29)	0.38
Body mass index (kg/m^2^)	27 ± 6	27 ± 6	28 ± 5	0.13
Papillary type, *n* (%)	134 (80)	99 (85)	35 (71)	0.05
Stage > pT 2, *n* (%)	77 (46)	54 (46)	23 (47)	0.93
Nodal involvement, *n* (%)	25 (15)	22 (18)	3 (6)	0.04
Extra-thyroidal uptake, *n* (%)	33 (20)	21 (18)	12 (24)	0.34
Nodal uptake, *n* (%)	7 (4)	7 (6)	0 (0)	0.08
Metastatic uptake, *n* (%)	13 (8)	7 (6)	6 (12)	0.17
Nodal and metastatic uptake, *n* (%)	13 (8)	7 (6)	6 (12)	0.17
Time interval surgery/therapy (days)	130 (19–131)	117 (22–1106)	162 (19–1314)	-
Administered ^131^I activity (MBq)	3700 (925–7400)	3700 (925–7004)	3700 (925–5920)	-
Serum Tg off LT4 (ng/mL)	6.7 (0.5–10,029)	6.2 (0.5–10,029)	8.8 (0.5–4476)	-
TSH off LT4 (mU/L)	60 (0.4–214)	68.2 (4–214)	47.8 (0.4–169)	-
Serum creatinine (mg/dl)	1 ± 0.2	1 ± 0.2	1 ± 0.3	0.51
Effective ^131^I half-life (hours)	16 ± 9	14 ± 7	20 ± 11	0.0001

Data are presented as mean ± SD, or median and range (minimum–maximum) or number and percentage (%). Tg: thyroglobulin; TSH: thyroid stimulating hormone.

**Table 2:** Multivariable linear regression analysis after backward variable selection to predict whole-body effective 131I half-life.

	β Coefficient	SE	*p*
Age	0.227	0.047	0.004
Male gender	0.128	1.563	0.085
Extra-thyroidal uptake	0.202	2.664	0.007
TSH off LT4	−0.148	0.018	0.055

SE: standard error; TSH: thyroid stimulating hormone.

**Table 3:** WBS results of the 11 patients for whom two consecutive sets of data concerning ^131^I whole-body retention are available.

	First ^131^I Treatment				Second ^131^I Treatment			
Pt	Tg (ng/mL)	TSH (mIU/L)	Effective ^131^I Half-Life (h)	Uptake at First Wbs	Tg (ng/mL)	TSH (mIU/L)	Effective ^131^I Half-Life (h)	Uptake at Second WBS
1	1000	0.4	42.6	Latero-cervical nodes, diffuse uptake on lungs, rib	3891	44	9.8	Latero-cervical nodes, diffuse uptake on lungs, humerus
2	40	3.8	36.6	Anterior neck	7	42	13	Anterior neck
3	434	7	32.5	Anterior neck, latero-cervical nodes, lung, mediastinum, rachis	156	35.6	19.6	Anterior neck, diffuse uptake in lung
4	3740	4.1	43.5	Anterior neck, diffuse uptake on lungs	2985	76	23.1	Anterior neck, latero-cervical nodes, diffuse uptake on lungs
5	63	35.6	23.1	Anterior neck	8.5	43.9	10.5	Anterior neck
6	10,029	40.2	18.5	Anterior neck, diffuse uptake on lungs, femur	4321	60.5	19.7	Diffuse uptake on lungs, femur
7	209	76.3	14	Anterior neck, latero-cervical node, diffuse uptake on lungs	115	184	13.3	Anterior neck, diffuse uptake on lungs
8	82	187	24.9	Anterior neck, mediastinum, lung	40	135.5	14	Mediastinum, lung
9	432	55.3	12.9	Anterior neck, latero-cervical node, diffuse uptake on lungs	0.5	66.6	9.3	Anterior neck, latero-cervical nodes
10	4476	88.7	10	Anterior neck, rachis, hip	573	64	11.1	Anterior neck, rachis, hip
11	919	159	29	Superior-mediastinum	444	140.7	17.5	No uptake

Tg: thyroglobulin; TSH: thyroid stimulating hormone; WBS: whole-body scan.

## Data Availability

The data presented in this study are available on request from the corresponding author. The data are not publicly available due to privacy restrictions.

## Abbreviations

RAI	Radioactive ^131^I
DTC	Differentiated thyroid cancer
TSH	Thyroid-stimulating hormone
rhTSH	Recombinant human TSH
Tg	Serum thyroglobulin
Tg-Ab	Tg-antibody
WBS	Whole-body scan
